# Promoting distributed photovoltaic adoption: An evolutionary game model approach for stakeholder coordination

**DOI:** 10.1371/journal.pone.0302241

**Published:** 2024-06-21

**Authors:** Biao Tao, Can Wang

**Affiliations:** 1 School of Electrical and Electronic Information Engineering, Hubei Polytechnic University, Wuhan, China; 2 School of Economics and Management, Wuhan University, Wuhan, China; Zhejiang Gongshang University, CHINA

## Abstract

Distributed photovoltaic (DPV) is a promising solution to climate change. However, the widespread adoption of DPV faces challenges, such as high upfront costs, regulatory barriers, and market uncertainty. Addressing these barriers requires coordinating the interests of stakeholders in the promotion of DPV. Therefore, this paper constructs a three-party evolutionary game model in a social network with the government, investment companies and residents as the main subjects and examines the influence of different subjects’ behavioral strategies on the promotion of DPV under the social learning mechanism. The results show that: (1) In the game equilibrium, both the government and residents hold a positive attitude towards the promotion of DPV; (2) Companies will obtain most of the subsidies through market power and information differences, resulting in the increase of government subsidies that do not always benefit residents; (3) The increase of energy consumption and pollution prevention costs can promote companies’ investment in DPV; (4) The increase of environmental protection taxes to a certain extent helps companies to take responsibility for promoting DPV, reducing the pressure on the government to promote it and increasing residents’ income. This study provides insights into the sustainable development of DPV.

## 1 Introduction

Renewable energy has become increasingly popular as a sustainable alternative to traditional energy sources, which are finite and contribute to climate change [1]. Photovoltaic (PV) technology has emerged as one of the most promising renewable energy sources, as it generates electricity using solar energy with minimal greenhouse gas emissions, contributing to climate change mitigation [2,3]. When PV energy is combined with distributed systems, known as distributed photovoltaic (DPV), it allows for electricity generation at or near the point of consumption. This can reduce energy transmission losses, enhance energy efficiency, and diversify the energy supply [4,5]. At the same time, DPV decentralizes energy production, which means that even if there is an outage or failure in one area, power can continue to be supplied in other areas [6]. This enhances the energy system’s resilience, reduces the risk of blackouts, and promotes energy decentralization, reducing reliance on large central power stations and traditional energy systems [7,8]. It is crucial for establishing a resilient energy system and improving energy availability. According to the International Energy Agency, DPV systems are expected to grow significantly in the coming years. By 2024, global capacity is expected to reach 600 GW, while household DPV installations are expected to double to about 100 million homes. This growth is driven by factors such as declining costs of PV technology, favorable policies and regulations, and increasing consumer demand for clean energy [9–11]. The widespread adoption of DPV can contribute to a more sustainable and resilient energy system and help address the urgent issue of climate change [12].

However, the adoption of DPV has been slow in some regions (for example, large markets such as China and the United States) despite the potential benefits [13]. There are several obstacles to spreading DPV that need to be addressed. One major challenge is the high upfront cost of installing DPV systems, which can deter some consumers and businesses from adopting the technology [14]. Additionally, the lack of standardized regulations and policies, particularly at the local level, can create uncertainty and hinder investment in DPV systems [15]. Technical barriers such as grid integration and variability in DPV output can also pose challenges to widespread adoption [16]. Finally, public awareness and understanding of the benefits of DPV can be limited, which can slow the uptake of the technology [17]. Currently, there are oppositions in society due to overloading in urban and non-urban spaces through large, medium, and small projects [18,19]. With limited land resources, some people object to using agricultural land or natural environments for solar panel installations because of the potential negative impacts on ecosystems and agriculture [20,21]. In addition, land occupation, project noise, and traffic problems associated with the construction of large-scale PV projects may cause inconvenience to the community’s quality of life [22]. Despite these objections and challenges, many local governments and energy companies are working hard to address them, such as selecting appropriate land uses, conducting environmental assessments, improving the sustainability of solar panels, and working closely with communities [23,24]. PV energy is still a key solution in reducing carbon emissions and driving the transition to renewable energy. Further promotion of DPV to realize its full potential as a sustainable energy source requires collaborative planning to balance stakeholders’ interests in the DPV promotion process [25].

From the perspective of goals and responsibilities, the main stakeholders of DPV promotion include the government, enterprises, and residents [26,27]. The government, investment enterprises, and residents all play crucial roles in adopting and promoting DPV technology. The government can provide incentives and regulations, investment enterprises can provide financing and business models, and residents can provide the demand for and installation of DPV systems. The game theory approach allows one to examine the interactions between multiple agents since each has its own goals and constraints [28,29]. Evolutionary game theory is a classical approach that combines game theoretic analysis and dynamic evolutionary analysis. It holds that individuals continuously adjust their behavior through imitation and learning, thus evolving optimal strategies in repeated games [30]. In DPV promotion, different stakeholders have different goals, resources, and strategies, and the interaction between them is dynamic and complex, adjusting according to the behavior of other stakeholders and environmental changes. By modeling the decision-making process and strategy updating mechanism of these stakeholders, the evolutionary game approach, can effectively capture the dynamic interactions and game processes among them and simulate how they learn, adapt, and adjust their strategies in a changing environment. This method not only realistically reflects reality but also provides rich dynamic insights [31]. Therefore, we believe the evolutionary game approach based on stakeholder coordination is suitable for modeling the dynamic evolution of DPV adoption promotion.

This paper proposes a tripartite evolutionary game model, which considers the government, investment enterprises, and residents as the main agents and examines how their decisions and interactions affect the promotion of DPV technology. The model considers the evolution of the agents’ strategies over time and how they adapt to changes in the social network. Further, we also consider the impact of social learning on decision-making. Social learning is a process in which individuals or groups obtain relevant environmental information and change their behaviors by following or observing the behaviors or results of others [32,33]. The social learning mechanism enables the agents in DPV promotion to learn from each other and adjust their strategies accordingly [34].

Compared to the existing literature, this paper contributes in the following aspects: (1) This study constructs a tripartite evolutionary game model incorporating the government, investment enterprises, and residents, enabling a more comprehensive examination of the dynamic interactions and decision changes among multiple stakeholders in the promotion process of DPV technology. This approach, more integrative and dynamic than the commonly focused single stakeholder or static analysis in existing literature, offers a novel perspective for understanding the complexities of DPV technology promotion. (2) This study explores the impact of the social learning mechanism on DPV technology promotion by modeling interactions and connections within a social network. The inclusion of this mechanism allows the model to not only capture the evolutionary process of individual strategies but also reflect the influence of changes in the social network environment on individual decision-making. This aspect, less elaborately discussed in existing literature, provides a significant theoretical foundation for a deeper understanding of the driving forces and obstacles to DPV technology promotion within a dynamic social network context. (3) This study reveals the complex impacts of key factors such as government subsidies, energy consumption and pollution prevention costs, and environmental protection taxes on DPV promotion, offering profound insights for the formulation of more effective DPV promotion policies.

The paper is structured as follows. The subsequent section is the Literature review, which reviews the existing research related to the promotion of DPV and presents the theoretical framework of this paper. Next, the Three-party game model section constructs a three-party evolutionary game model for DPV promotion and analyzes the subject interests. Then, the Social learning section introduces the social learning mechanism in the process of DPV promotion. Further, the Simulation section analyzes the model simulation results and performs sensitivity analysis on key parameters. Finally, the Conclusions and implications section summarizes the main conclusions of this paper and provides management implications for policy makers.

## 2 Literature review

Due to the important contribution of renewable and green energy in mitigating the climate change problem, a wide range of literature explores how to promote their development. [35] explored the energy development patterns in Pakistan and identified several key obstacles to developing renewable energy. These obstacles include policy, regulatory, institutional, financial, economic, market, technological, and information and social barriers. [36] identified 22 obstacles to renewable energy development in Nepal, categorizing them into six types: social, policy and political, technological, economic, administrative, and geographical. [37] found that the success of renewable energy in the African region is constrained by various factors, including weak institutional frameworks and infrastructure, high initial capital costs, limited dissemination strategies, a lack of skilled manpower, inadequate baseline information, and weak maintenance services. An analysis of the achievements and challenges in renewable energy development in India identified various obstacles faced by the renewable energy industry [38]. These obstacles encompass not only the major barriers proposed by [35] but also include awareness, education, training, and environmental obstacles. Through a systematic review, factors influencing societal acceptance and stakeholder perspectives on hydrogen-related technologies were identified [39]. These factors include prior knowledge, perceived costs/risks, environmental awareness, higher education and income, individual and distributional interests, infrastructure availability, and proximity to hydrogen facilities. [40] summarized the determinants affecting green energy adoption in four areas through a comprehensive literature survey: technological issues, adopter level, business promotion, and environmental challenges. In summary, scholars have extensively researched the factors and barriers affecting the development of renewable and green energy. It can be observed that there are numerous influencing factors, and there is no unified framework. Scholars often employ various methods and perspectives based on their research objectives for analysis.

As an advanced renewable energy technology, existing research has primarily identified the key factors and significant barriers influencing the adoption and promotion of DPV. Similarly, a diverse range of factors influence the development of DPV. Based on a survey of the Chinese DPV electricity market and policy stakeholders, [41] identified the primary barriers to DPV installation, which include financial barriers for property owners, corporate ambivalence, complex ownership structures of buildings, and delays in government policy implementation. [42] identified key factors influencing consumer adoption of DPV technology from operators’ perspective. They found that electricity cost, generation capacity, and photovoltaic system cost are the most relevant indicators. [43,44] explored the primary driving factors for promoting distributed energy in China. Their studies indicated that infrastructure investment, technological advancement, external oil dependency, energy consumption structure, energy subsidies, international oil prices, and urbanization are the major factors influencing the promotion of distributed energy, and their impacts vary across different regions. [45] explored the influencing factors of DPV development in Argentina, and the results indicated that environmental factors such as political instability, energy subsidies, and high inflation rates were considered as the primary obstacles. [46] conducted a questionnaire survey involving 430 participants in Poland and found that factors influencing consumers’ attitudes towards PV technology are related to environmental concerns, economic factors, and factors associated with the usability of PV technology. [47] conducted a survey involving 20 experts and 29 electricity consumers and found that consumers primarily focus on generation capacity, electricity costs, photovoltaic system costs, and financial capability when adopting distributed photovoltaic electricity generation.

Among these many influences, previous literature has identified government subsidies as the most important factor affecting DPV diffusion, as they can help reduce costs to increase the investment’s profitability [48–50]. However, recent literature has noted that the focus is on social acceptance [51,52]. A sample of 1424 questionnaires was collected to investigate residents’ willingness to install DPV equipment in typical regions of China, and the results showed that living conditions, cost, installation risk, maintenance risk, and economic benefits were the five most significant influencing factors [53]. [54] used questionnaire data from 400 Chinese participants in Zhejiang Province to examine the effects of environmental concerns and innovation on the intention to use household PV systems among Chinese residents and showed that both environmental concerns and the ability to innovate were positively related to the intention to use. These studies show that the government as the promoter and the residents as the participants in accepting these technologies are the two most important actors in promoting DPV. In addition, stakeholders such as enterprises, PV cell suppliers, and banks also make important contributions to the promotion of DPV [55].

Based on this, scholars are increasingly inclined to study the promotion of the DPV from the perspective of stakeholders, categorizing the main factors affecting the promotion of the DPV into aspects related to stakeholders (government, enterprises, and residents). [50] used evolutionary game theory and empirical analysis to analyze the interaction between the government and users in DPV installation in China. The results show that the effect of subsidy policy is constrained by regional economic development, and the government should choose a high or low subsidy strategy according to local conditions to reasonably guide the development of the PV industry. [56] reveal the impact of mixed policies of incentives and penalties on residents’ decision-making and institutional evolution by developing an evolutionary game model between the government and residents to provide a theoretical explanation for promoting the sustainable application of residential DPV generation in China. The results show that using a combination of dynamic subsidies and static taxes is the most effective and feasible policy option to stabilize residential and governmental PV development patterns. [55] developed a three-party evolutionary game model of government, firms, and households to analyze stakeholders’ strategies for promoting DPV in China. The results show that increasing initial willingness, financial subsidies, punitive taxes, and operational efficiency coefficients have positive effects on encouraging participants to promote DPV, while additional costs and investment costs have negative effects on participants’ promotion of DPV. [57] constructed an evolutionary game model of the government, investors, and PV firms to explore the path of PV building promotion under different scenarios.

These studies conducted from the stakeholders’ perspective have inspired this paper. However, they only considered the strategic choices of stakeholders based on maximizing their interests. They did not consider the effect of intra-group interaction, i.e., the influence of social learning mechanisms. Studies have long found that neighborhood peer effects increase PV adoption [58,59]. An effective social learning process can improve public understanding of residential PV installations and better inform decision-making [60]. [61] noted that the peer effect in DPV adoption typically comes from peers with a DPV user experience who confirm that the technology works as expected and without unexpected issues. On this basis, [34] investigated a large-scale behavioral intervention aiming to actively use social learning and peer interaction to encourage the adoption of residential solar PV systems. Therefore, this paper builds on existing research by incorporating social learning mechanisms into an evolutionary game model and examining the impact of social learning on stakeholders’ DPV promotion strategies.

**Fig 1:**
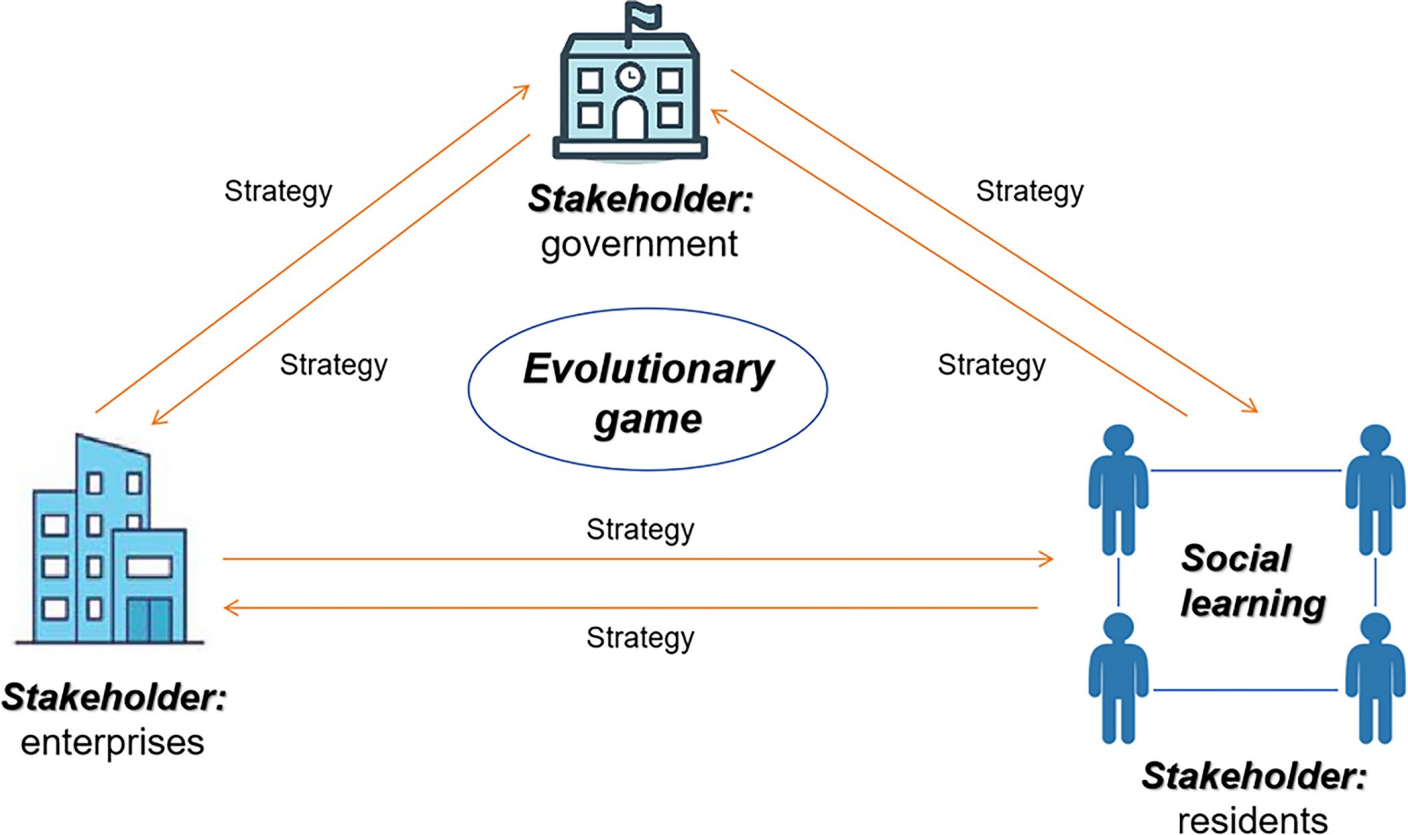
The theoretical framework for promoting DPV adoption.

In summary, this paper constructs a theoretical framework for promoting DPV adoption by integrating the multiple perspectives of stakeholders, the dynamics of evolutionary game theory, and the interactive mechanisms of social learning theory. This framework elucidates the interplay, strategic adaptations, and influence of social learning among stakeholders, represented by government, investment enterprises, and residents, in promoting DPV. It establishes a theoretical foundation for comprehending and interpreting the dynamic process underlying the diffusion of DPV. The theoretical framework is shown in Fig 1.

First, the multi-stakeholder perspective helps to clarify the different actors in the process of DPV promotion, i.e., the government, investing enterprises and residents, as well as their respective interests and objectives. The government typically focuses on policy formulation, financial support, and environmental and societal benefits, while enterprises primarily prioritize market competition and profitability, and residents consider economic costs and potential gains. This multifaceted drive shapes their decisions and actions in the promotion of DPV.

Second, the dynamic mechanism of evolutionary game theory is key to analyzing and understanding the changes in stakeholder behaviors and their interactions in the process of promoting DPV technology. The evolutionary game theory model considers temporal dynamics and the adaptive strategies of participants over time, emphasizing that the strategic choices of individuals or groups are dynamically adaptive and evolve in a constantly changing environment [30]. The strategy update rules in evolutionary games describe how stakeholders adjust their strategies based on previous experiences and changes in the surrounding environment [31]. This implies that throughout the evolution process, stakeholders dynamically adjust their strategies based on changes in the environment and the strategies of other stakeholders, directly impacting the proliferation and development of DPV.

Finally, social learning theory underscores the process through which individuals or groups acquire relevant environmental information and alter their behaviors by observing or imitating the actions or outcomes of others [62]. Within the framework of DPV promotion, the social learning mechanism allows stakeholders to learn from each other’s experiences through neighborhood effects, information dissemination, or sharing of experiences, drawing lessons from both successful and failed cases, and accordingly adjusting their promotion strategies and behaviors [34]. This interactive mechanism deepens the understanding of information flow, knowledge sharing, and group behavior patterns within social networks, providing significant support for effectively disseminating DPV technology.

In summary, this theoretical framework offers a novel perspective for a comprehensive analysis of the complexities, dynamics, and multidimensionality of DPV promotion, elucidating the fundamental principles and mechanisms of stakeholder interactions in the process of facilitating DPV and furnishing theoretical foundations and practical guidance for designing effective DPV promotion strategies and practices.

## 3 Three-party game model

### 3.1 Model description and assumptions

In the promotion of distributed PV, the government, investing enterprises, and residents constitute the three main stakeholders [63]. Their behaviors are influenced by each other’s interactions. First, the government plays the role of a guide and regulator. The government formulates policies and regulations, such as tax incentives and subsidy programs, to encourage the widespread use of distributed PV [64,65]. Government decisions have a direct impact on the behavior of investing enterprises and residents, as government policies will determine whether they will receive sufficient incentives to participate in PV projects. Second, as key participants in distributed PV projects, investment enterprises bear the responsibility for the development, operation, and maintenance of the projects [66]. They expect to receive a substantial return on electricity sales. However, the usual production activities of the investing enterprises may have negative impacts on the environment. These are usually not accounted for in the costs of the enterprises but are borne by society and the environment [67]. Finally, residents are also important players as resource providers and consumers of electricity. They can provide vacant sites such as rooftops and carports for the installation of PV panels. As partners of the investing enterprises, they can share the revenue from electricity sales with the enterprises.

**Fig 2:**
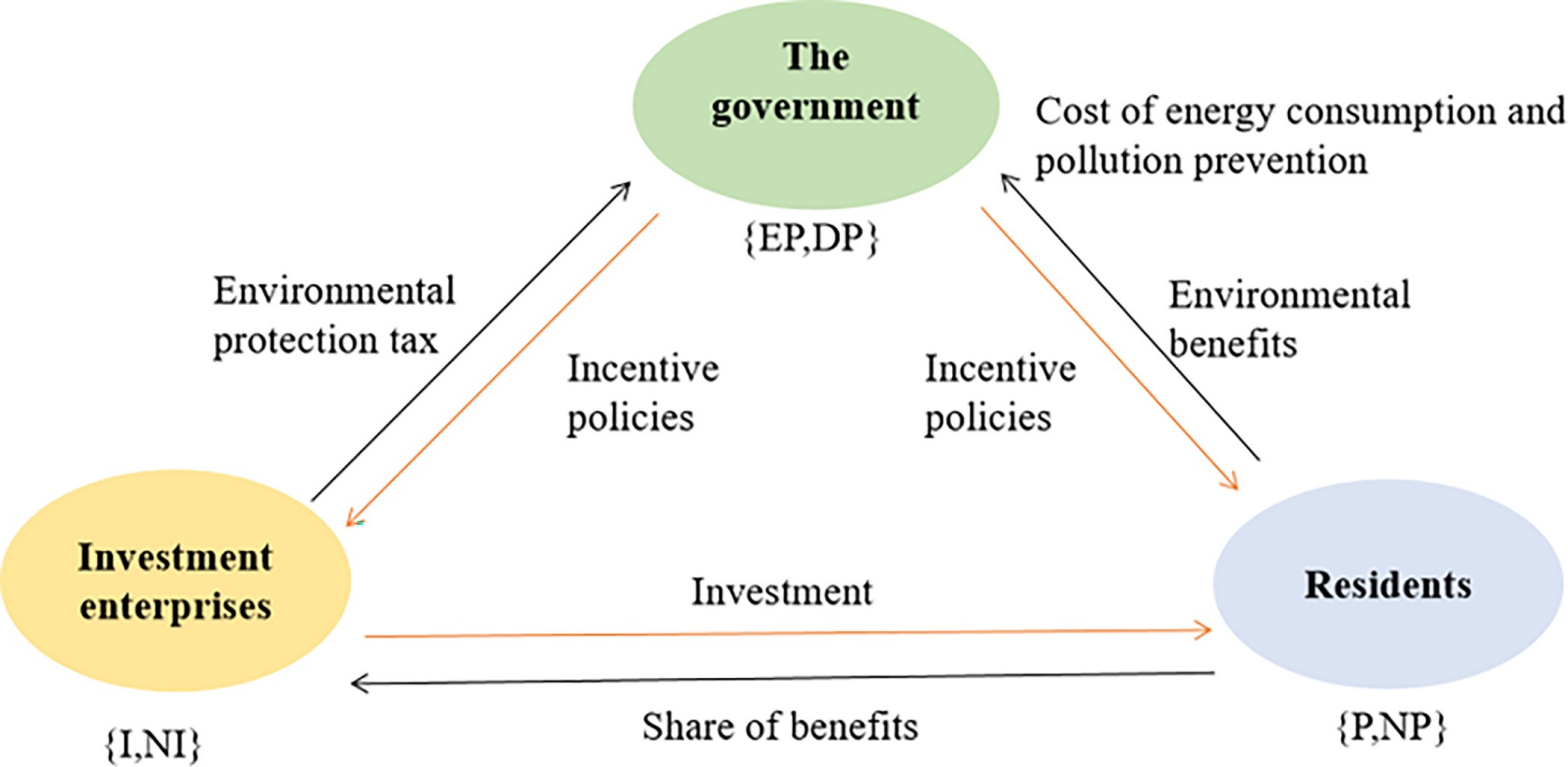
The interest relationship among the of three subjects.

Therefore, this paper constructs a multi-agent game model based on the government, investment enterprises, and residents. **Fig 2** shows the interesting relationship among the three. The government regulates the relationship between stakeholders by formulating policies and regulations to promote the development of DPV. Influenced by government policies, investment enterprises gain revenue by participating in DPV projects and take some responsibility for environmental protection. Residents participate in the project by providing vacant resources, obtaining certain economic gains, and contributing to the improvement of the social environment. This cooperative mechanism not only reduces greenhouse gas emissions but also creates employment opportunities and promotes sustainable economic and social development.

Based on the above analysis, we set the following assumptions:

**Assumption 1**: The government, investing enterprises, and residents are all finite and rational. In real life, it is difficult for individuals to be completely rational when making decisions, and they gradually get a stable strategy by constantly adjusting their behavioral strategies over a long period, i.e., each subject realizes the equilibrium through a complex interactive dynamic game.

**Assumption 2**: The government, investment enterprises, and residents all have two game strategies. The government’s strategy set is {EP, DP}, where EP indicates encouraging the promotion of DPV, and DP indicates discouraging the promotion of DPV. The investment enterprise’s strategy set is {I, NI}, where I indicates investment in DPV and NI indicates no investment in DPV. The resident’s strategy set is {P,NP}, where P indicates provide site, NP indicates do not provide site.

**Assumption 3**: When the government encourages the promotion of DPV but the investment enterprise chooses not to invest, the enterprise has to pay a certain amount of environmental protection tax to the government. This measure internalizes the environmental costs of the enterprise and makes the enterprise responsible for the environmental impacts of its actions.

**Assumption 4**: When the investment enterprise chooses not to invest, and the resident chooses not to provide the site, the government needs to pay to address the cost of energy consumption and pollution prevention. This measure reflects the Government’s responsibility for environmental protection.

Based on the previous model description, the government’s subsidies to investment enterprises and residents satisfy the following relationships:

$$f_{4}=\alpha g_{2}$$ (1)

$$u_{3}=(1-\alpha )g_{2}$$ (2)

And the distribution of electricity sales revenue between investment enterprises and residents follows the following relationships:

$$f_{1}=\beta f$$ (3)

$$u_{1}=(1-\beta )f$$ (4)

In Eq (1) to Eq (4), *f* denotes the electricity benefit. *f*_1_ denotes the electricity benefit of an investment enterprise. *f*_4_ denotes government subsidies obtained by an investment enterprise. *u*_1_ denotes the electricity benefit of a resident. *u*_3_ denotes government subsidies obtained by a resident. *g*_2_ denotes government subsidies. *α* denotes the percentage of government subsidies to an investment enterprise, and β denotes the percentage of electricity benefits captured by the investment enterprise.

### 3.2 Analysis of subjective interests

There are two strategies for different subjects. Considering that the government is the policy maker and the leader of DPV promotion. Based on this, we divide the game into two categories: (1) the government encourages the promotion of DPV; (2) the government discourages the promotion of DPV.

#### 3.2.1 The government encourages the promotion of DPV

When the government encourages the promotion of DPV, a certain subsidy will be given to the resident or investment enterprise. The benefit matrix of the three stakeholders is shown in Table 1.

**Table 1:** Revenue matrix for the resident and investment enterprise under EP.

		The resident	
		P	NP
**Investment enterprise**	I	*g*_1_−*g*_2_; *f*_1_−*f*_2_+*f*_4_; *u*_1_−*u*_2_+*u*_3_	*g*_1_−*g*_2_; *f*_1_−*f*_2_+*f*_4_+*u*_1_−*u*_2_+*u*_3_; 0
	NI	*g*_1_−*g*_2_+*f*_3_; −*f*_3_; *u*_1_−*u*_2_+*u*_3_+*f*_1_−*f*_2_+*f*_4_	−*g*_3_+*f*_3_; −*f*_3_; 0

In Table 1, *g*_1_ denotes environmental benefits to the government. *g*_2_ denotes government subsidies. *g*_3_ denotes government costs to address energy consumption and pollution prevention. *f*_1_ denotes the electricity benefit of an investment enterprise. *f*_2_ denotes the investment costs of an investment enterprise. *f*_3_ denotes the environmental protection tax. *f*_4_ denotes government subsidies obtained by an investment enterprise. *u*_1_ denotes the electricity benefit of a resident. *u*_2_ denotes site fees. *u*_3_ denotes government subsidies obtained by a resident.

Table 1 contains four scenarios.

**Scenario 1**: The government encourages the promotion of DPV, the enterprise invests in DPV, and the resident provides the site, i.e. {EP,I,P}.

For the government, the basic benefit is mainly the environmental benefit, denoted as *g*_1_. The cost is the subsidy *g*_2_ paid to the resident and the enterprise. Therefore, the government’s utility is *g*_1_−*g*_2_. For the enterprise, the basic benefits are the electricity benefit *f*_1_ and government subsidies *f*_4_. The cost is the investment cost paid *f*_2_. Therefore, the enterprise’s utility is *f*_1_−*f*_2_+*f*_4_. For the resident, the basic benefits are electricity benefit *u*_1_ and government subsidies *u*_3_. The cost refers to the cost *u*_2_ for providing the site, so that the utility of the resident is *u*_1_−*u*_2_+*u*_3_.

**Scenario 2**: The government encourages the promotion of DPV, the enterprise invests in DPV, but the resident does not provide the site, i.e. {EP,I,NP}.

For the government, the utility is similar to Scenario 1 and is denoted as *g*_1_−*g*_2_. For the enterprise, in addition to *f*_1_−*f*_2_+*f*_4_, it needs to pay the site fee *u*_2_, but it can obtain the electricity benefit *u*_1_ and government subsidy *u*_3_. Therefore, the utility of the firm is *f*_1_−*f*_2_+*f*_4_+*u*_1_−*u*_2_+*u*_3_. For the resident, there is no benefit and no cost because no site is provided, so the resident’s utility is 0.

**Scenario 3**: The government encourages the promotion of DPV, the enterprise does not invest in DPV, and the resident provides the site, i.e. {EP,NI,P}.

For the government, different from Scenario 3, the government also gets the environmental protection tax *f*_3_ paid by the enterprise, so the utility of the government is *g*_1_−*g*_2_+*f*_3_. For the enterprise, it has to pay the environmental protection tax *f*_3_ to the government. Therefore, the enterprise’s utility is −*f*_3_. For the resident, in addition to *u*_1_−*u*_2_+*u*_3_, it needs to pay an additional cost *f*_2_ due to the enterprise’s disinvestment, but it can obtain the electricity benefit *f*_1_ and government subsidy *f*_4_. Therefore, the utility of the firm is *u*_1_−*u*_2_+*u*_3_+*f*_1_+*f*_2_+*f*_4_.

**Scenario 4**: The government encourages the promotion of DPV, the enterprise does not invest in DPV, and the resident does not provide the site, i.e. {EP,NI,NP}.

For the government, its benefit is mainly the environmental protection tax *f*_3_ paid by enterprises, and its cost is the cost *g*_3_ of addressing energy consumption and pollution prevention. Therefore, the government’s utility is −*g*_3_+*f*_3_. For the enterprise, the utility is similar to scenario 3 and is denoted as −*f*_3_. For the resident, the utility is similar to scenario 2 and is denoted as 0.

**3.2.2 The government discourages the promotion of DPV.** When the government does not encourage the promotion of DPV, it will not give a certain amount of subsidies to the residents or investment enterprises. The benefit matrix of the three stakeholders is shown in Table 2.

**Table 2:** Revenue matrix for the resident and investment enterprise under DP.

		The resident	
		P	NP
**Investment enterprise**	I	*g*_1_; *f*_1_−*f*_2_; *u*_1_−*u*_2_	*g*_1_; *f*_1_−*f*_2_+*u*_1_−*u*_2_; 0
	NI	*g*_1_; 0; *u*_1_−*u*_2_+*f*_1_−*f*_2_	−*g*_3_; 0; 0

In Table 2, *g*_1_ denotes environmental benefits to the government. *g*_3_ denotes government costs to address energy consumption and pollution prevention. *f*_1_ denotes the electricity benefit of an investment enterprise. *f*_2_ denotes the investment costs of an investment enterprise. *u*_1_ denotes the electricity benefit of a resident. *u*_2_ denotes site fees.

Table 2 contains the following four scenarios.

**Scenario 1**: The government discourages the promotion of DPV, the enterprise invests in DPV, and the resident provides the site, i.e. {DP,I,P}.

For the government, no subsidies are required to be paid to residents or enterprises, and the basic benefit is mainly the environmental benefit, denoted as *g*_1_. Therefore, the government’s utility is *g*_1_. For the enterprise, the basic benefit is mainly the apportioned benefit from the sale of electricity, denoted as *f*_1_. The cost is the investment cost paid *f*_2_. Therefore, the enterprise’s utility is *f*_1_−*f*_2_. For the resident, the basic benefit refers mainly to the apportioned benefit from the sale of electricity, recorded as *u*_1_. The cost refers to the cost *u*_2_ for providing the site so that the utility of the resident is *u*_1_−*u*_2_.

**Scenario 2**: The government discourages the promotion of DPV, the enterprise invests in DPV, and the resident does not provide the site, i.e. {DP,I,NP}.

For the government, the utility is similar to scenario 1 and is denoted as *g*_1_. For the enterprise, in addition to *f*_1_−*f*_2_, it needs to pay the site fee *u*_2_, but it can obtain the electricity benefit *u*_1_. Therefore, the utility of the firm is *f*_1_−*f*_2_+*u*_1_−*u*_2_. For the resident, there is no benefit and no cost because no site is provided, so the resident’s utility is 0.

**Scenario 3**: The government discourages the promotion of DPV, the enterprise does not invest in DPV, and the resident provides the site, i.e. {DP,NI,P}.

For the government, the utility is similar to scenario 1 and is denoted as *g*_1_. For the enterprise, it does not receive any revenue for not investing and does not have to pay the environmental protection tax, Therefore, the enterprise’s utility is 0. For the resident, in addition to *u*_1_−*u*_2_, it needs to pay an additional cost *f*_2_ due to the enterprise’s disinvestment, but it can obtain the electricity benefit *f*_1_. Therefore, the utility of the firm is *u*_1_−*u*_2_+*f*_1_−*f*_2_.

**Scenario 4**: The government discourages the promotion of DPV, the enterprise does not invest in DPV, and the resident does not provide the site, i.e. {DP,NI,NP}.

For the government, it pays *g*_3_ for energy consumption and pollution prevention. Therefore, the government’s utility is −*g*_3_. For the enterprise, the utility is similar to scenario 3 and is denoted as 0. For the resident, the utility is similar to scenario 2 and is denoted as 0.

The above game matrix is only for a single resident, this paper assumes that there are, *m* residents, where *m*_1_ residents choose to provide the site and *m*_2_ residents choose not to provide the site, then the utility of the government and enterprise is shown in Table 3.

**Table 3:** Revenue matrix for the government and investment enterprise under DP.

		The enterprise	
		I	NI
**the government**	EP	*m*_1_(*g*_1_−*g*_2_)+*m*_2_(*g*_1_−*g*_2_); *m*_1_(*f*_1_−*f*_2_+*f*_4_) +*m*_2_(*f*_1_−*f*_2_+*f*_4_+*u*_1_−*u*_2_+*u*_3_)	*m*_1_(*g*_1_−*g*_2_+*f*_3_) +*m*_2_(−*g*_3_+*f*_3_); *m*_1_(−*f*_3_)+*m*_2_(−*f*_3_)
	DP	*m*_1_(*g*_1_)+*m*_2_(*g*_1_); *m*_1_(*f*_1_−*f*_2_)+*m*_2_(*f*_1_−*f*_2_+*u*_1_−*u*_2_)	*m*_1_(*g*_1_)+*m*_2_(−*g*_3_); 0

## 4 Social learning

Social learning in social networks refers to the process in which individuals or groups acquire knowledge and experience through observation, imitation, and exchange of information with others in a network composed of various relationships and interactions [30,31]. Social learning occurs within social networks, influenced by social relationships, where individuals in the network make decisions based on their goals and environment [32]. This process involves interactions among the government, investment companies, and residents. Therefore, the model proposed in this paper can be considered as an agent-based model.

**Fig 3:**
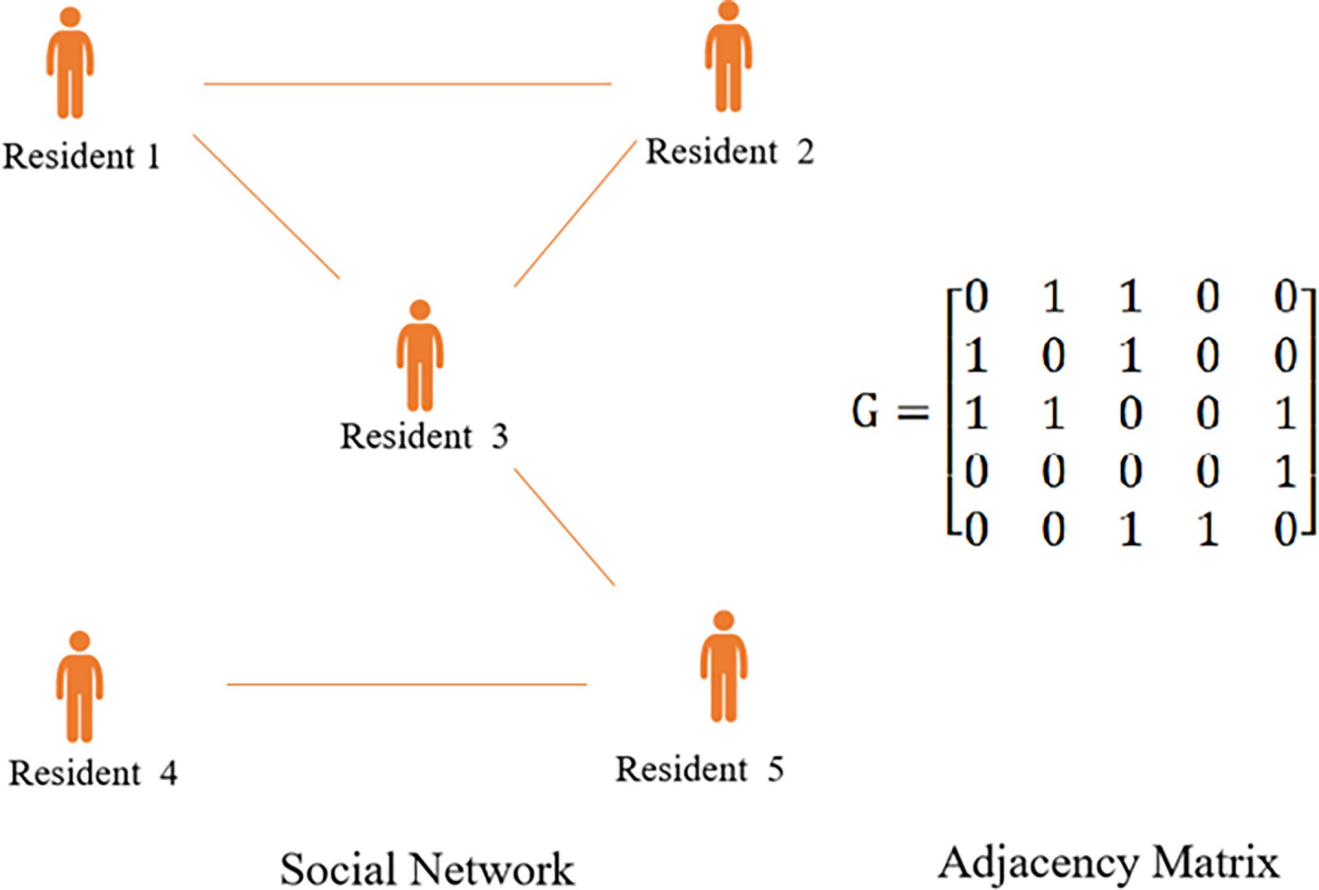
Social network among residents.

Assume that *m* residents occupy the network vertices. Since the residents’ communication is bidirectional, each interaction between two residents can be represented by an undirected edge, as shown in Fig 3. The network of *m* residents can be written as a symmetric matrix $G={\left(g_{ij}\right)}_{m\times m}=\left[\begin{array}{cccc} g_{11} & g_{12} & \cdots & g_{1m}\\ g_{21} & g_{22} & \cdots & g_{2m}\\ \vdots & \vdots & \vdots & \vdots \\ g_{m1} & g_{m2} & \cdots & g_{mm} \end{array}\right]$, where *g*_ii_ = 0,*g*_*ij*_ = *g*_*ji*_, *i*,*j* = 1,2,⋯,*m*.*g*_*ij*_ = 1 means that there is social communication between resident *i* and resident *j*.*g*_*ij*_ = 0 means that there is no social communication between resident *i* and resident *j*. *N*_*i*_ = {*j*|*g*_*ij*_ = 1} denotes the neighbor of resident i, i.e., the learning object of resident *i*.

Residents can communicate with each other to learn each other’s strategies, and update their own strategies by comparing multiple strategies. The details are as follows: the resident communicate with its neighbors and obtains the strategies of all neighbors. Note that *n*_*i*1_ expresses the number of neighbors who choose to provide a site, *s*_*i*1_ expresses the utility of a neighbor who chooses to provide a site, *n*_*i*2_ expresses the number of neighbors who choose not to provide a site, and *s*_*i*2_ expresses the utility of a neighbor who chooses not to provide a site. Then the probability that resident *i* chooses to provide a site can be denoted as.

$$p_{i1}=\frac{1}{1+exp(\beta (w_{2}-w_{1}))}$$ (5)

$$w_{1}=\beta_{1}\frac{n_{i1}}{n_{i1}+n_{i2}}+\beta_{2}\frac{s_{i1}}{s_{i1}+s_{i2}}$$ (6)

$$w_{2}=\beta_{1}\frac{n_{i2}}{n_{i1}+n_{i2}}+\beta_{2}\frac{s_{i2}}{s_{i1}+s_{i2}}$$ (7)

where *β*_1_+*β*_2_ = 1.*β*_1_ denotes the resident’s weight in favor of their neighbors’ strategies and *β*_2_ denotes the resident’s weight in favor of revenue. *w*_1_ and *w*_2_ denote the weighted evaluation results of the resident’s choice to provide the site and not to provide the site, respectively. Eq (5) refers to the probability that a consumer chooses to provide a site. The larger *w*_1_ and the larger *p*_*i*1_, the more likely the consumer chooses to provide the site. Eq (6) is the weighted average of the proportion of neighbors providing the site and the proportion of revenue. The larger the number of neighbors providing the site and their revenue, the larger *w*_1_. According to Eq (5), the probability that consumers are willing to provide the site increases. Similarly, Eq (7) shows that the larger *w*_2_ is, the lower the probability that the consumer is willing to provide the site.

## 5 Simulation

### 5.1 Parameter setting

The parameter values used in this paper are referenced from [54]. Considering the substantial financial investments required by investment enterprises in aspects such as the construction, operation, and maintenance of DPV projects, this paper sets the government’s subsidy ratio to investment enterprises and residents at 0.8 and 0.2, respectively. Similarly, the proportions of electricity benefits received by investment enterprises and residents are also set at 0.8 and 0.2, respectively. The values of different parameters are set as shown in Table 4.

**Table 4:** Parameter values.

Parameters	value(¥/year)	Parameters	value(¥/year)	Parameters	value(¥/year)
*g*_1_	[491.8,983.6]	*f*_1_	[8700,17400]	*u*_2_	[1000,2000]
*g*_2_	[116,232]	*f*_2_	[3747,7494]	*α*	0.8
*g*_3_	[550,1100]	*f*_3_	[1238.25,2476.5]	*β*	0.8

Currently, the installed capacity of distributed photovoltaic power stations in regions such as Shandong and Henan is roughly 10–20 kilowatts per household, covering an area of about 100 to 200 square meters, with an annual electricity generation ranging from 11,600 to 23,200 kilowatt-hours. The electricity generated by distributed photovoltaics encompasses the energy value and includes environmental value. This article uses the green certificate price to measure this environmental value, representing the environmental benefits (*g*_1_) the government can obtain. According to data from the China Energy Network, the trading prices of subsidy-free green certificates for photovoltaics in 2023 are concentrated in the range of 30 to 50 yuan per certificate, averaging an environmental benefit of 0.0424 yuan per kilowatt-hour. Therefore, the government’s environmental benefits can be set at 491.8 to 983.6 yuan per year.

According to the subsidy policies in 2023, there are variations in subsidies across different regions in China. For instance, the distributed photovoltaic subsidy in Hefei, Anhui, ranges from 0.2 to 0.4 yuan per kilowatt-hour, while in Guangdong, it is 0.01 yuan per kilowatt-hour, and in Xi’an, Shaanxi, it is 0.1 yuan per kilowatt-hour. On average, government subsidies are approximately 0.01 yuan per kilowatt-hour. Therefore, this article sets the government subsidy (*g*_2_) at 116–232 yuan per year. To assess the government cost of addressing energy consumption and pollution prevention issues, this article employs the baseline emission factor of China’s regional power grids. The annual electricity generation of distributed photovoltaic power stations ranges from 11,600 to 23,200 kilowatt-hours. If this electricity were generated using traditional energy sources, it would result in carbon emissions of 9.96 (11.6 MWh × 0.8587 tCO2/MWh) to 19.92 (23.2 MWh × 0.8587 tCO2/MWh) tons, where 0.8587 tCO2/MWh is the average emission factor of China’s regional power grids. Based on the average price of the Chinese carbon market in 2023, which is 55.3 yuan per ton, the government cost of addressing energy consumption and pollution prevention issues can be defined as 550–1100 yuan.

Currently, the benchmark grid-connected electricity price for photovoltaic power stations in China varies based on different resource areas, with an average price of 0.75 yuan per kilowatt-hour. Therefore, the electricity cost benefit for investing enterprises can be defined as 8,700–17,400 yuan per year. In 2022, the initial investment cost for distributed photovoltaic systems in China’s industrial and commercial sectors was 3.74 yuan/W, and it is expected to decrease to 3.42 yuan/W in 2023. Since detailed data for 2023 has not been released, this article uses 2022 as the baseline and converts the investment cost based on the average generation cost (20-year conversion), resulting in an investment cost for enterprises of 3,747–7,494 yuan per year.

According to the Implementation Regulations of the Enterprise Income Tax Law of the People’s Republic of China, the general corporate income tax rate is 25%. Therefore, this article sets the environmental protection tax for enterprises at 1,238.25–2,476.5 yuan per year. The land use fee is approximately 10–20 yuan per square meter. Hence, this article sets the land use fee that residents can receive at 1,000–2,000 yuan per year. All the mentioned parameters are obtained from the China Energy Network (https://www.china5e.com/). This article is written in Python 3.8 to create a simulation program. Various subject parameters are randomly selected during the simulation, all within above specified ranges.

### 5.2 Analysis of results

Based on the above parameter values, we conduct 300 simulations to reveal the impact of different decision-making behaviors of government, enterprises, and residents on DPV promotion.

**Fig 4:**
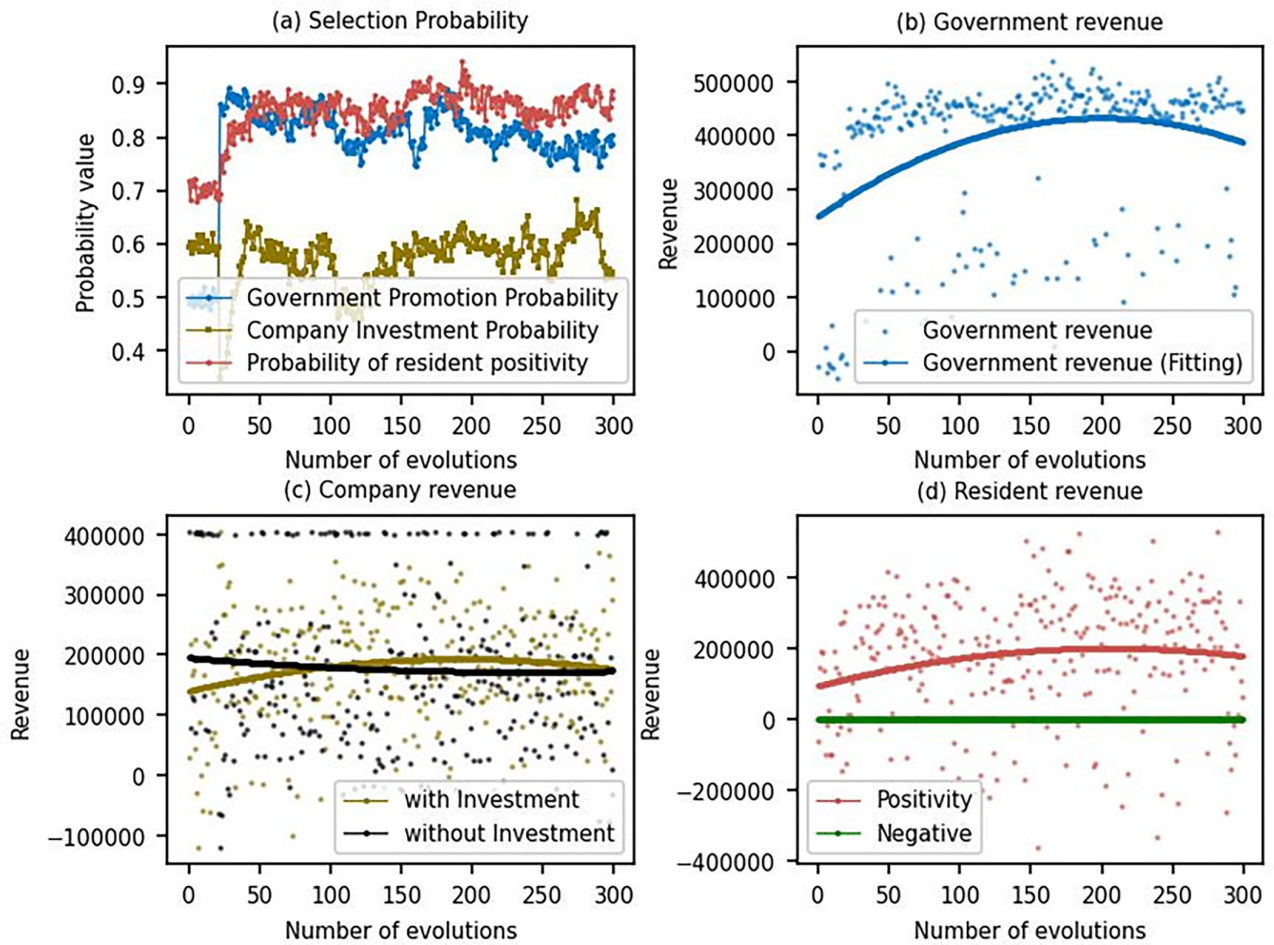
Three-party game results.

From Fig 4(A), as the number of evolutions increases, the government gradually encourages the promotion, and the selection probability stabilizes at around 0.8. This indicates that the government increasingly recognizes the importance of promoting DPV adoption. The number of residents choosing to offer sites is also gradually increasing and stabilizing at around 0.7, suggesting that residents are becoming more receptive to the government’s promotion efforts and are increasingly willing to participate in DPV initiatives. However, enterprises’ probability of choosing to invest or not to invest remains essentially the same, at around 0.5. This implies that enterprises are not significantly influenced by the government’s promotion efforts and remain uncertain about the benefits of investing in DPV projects. Fig 4(B) shows that the utility for both the government and residents is gradually increasing as the government gradually increases its promotion efforts. This demonstrates that the government’s encouragement of DPV adoption benefits itself and its residents. On the other hand, Fig 4(C) reveals no significant difference in the utility for enterprises from investing or not investing in DPV. This also means that government incentives to promote DPV do not interest enterprises. This lack of interest could be due to various factors, such as concerns about the return on investment, perceived risks associated with DPV projects, or the inability of government incentives to sufficiently address enterprises’ financial and operational challenges. From Fig 4(D), it can be observed that residents can increase their utility by choosing to provide the site for DPV projects, which further supports the conclusion that government promotion efforts are beneficial to residents. In conclusion, the government will actively promote DPV, and residents will respond positively. However, for enterprises, the positive strategies of the government and residents do not positively influence enterprises’ business strategy. This may indicate that the government needs to revise its promotional efforts to better address the concerns and interests of enterprises in order to ensure a more comprehensive and successful adoption of DPV technology across all sectors.

### 5.3 Sensitivity analysis

In order to further verify the effectiveness of the method, we explored the impact of different government subsidies *g*_2_, government costs to solve energy consumption and pollution prevention *g*_3_, environmental protection tax *f*_3_, and social learning effect *β* (Formula 5) on the decision-making results.

#### 5.3.1 The impact of different government subsidies *g*_2_

**Fig 5:**
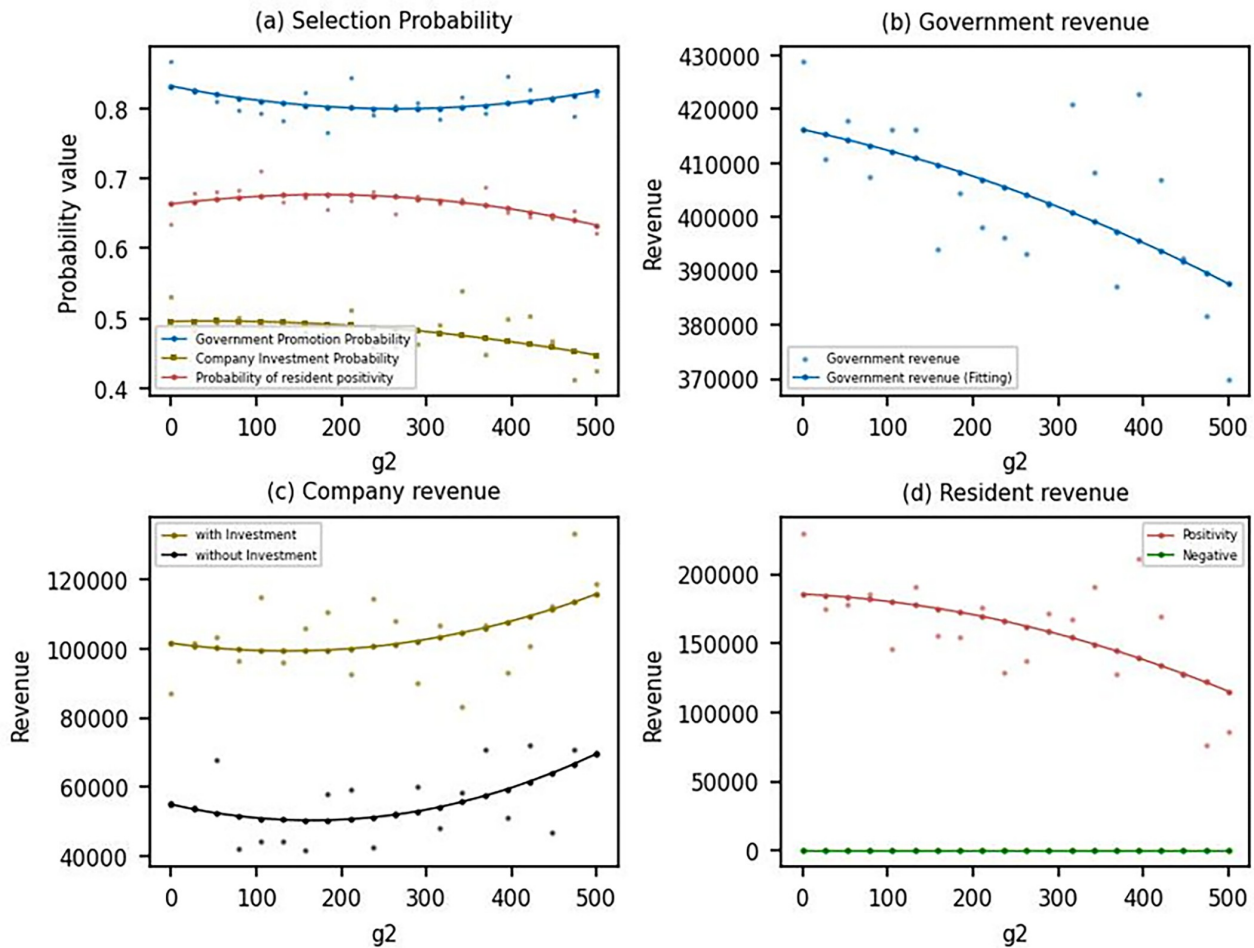
Evolutionary results under different *g*_2_.

As shown in Fig 5, it is evident that changes in government subsidies have not significantly altered the strategies of the government, businesses, or residents. However, they have had a noticeable impact on their utility (Fig 5(A)). When government subsidy expenditure increases from 400 to 1600, government utility gradually decreases from around 420,000 to 380,000 (Fig 5(B)), while the utility of both businesses, whether choosing to invest in the photovoltaic industry or not, increases by approximately 10,000 (Fig 5(C)). Government subsidies effectively support businesses in the photovoltaic market; however, this comes at the cost of sacrificing government resources. The government needs to consider the efficient allocation of resources to ensure that it does not overly rely on subsidies. Therefore, the government should seek more effective ways to support the industry, such as technological innovation or market regulation, to reduce its dependence on fiscal resources. The increase in utility for businesses stems from the subsidy policy reducing risks and costs. Government subsidies can alleviate the capital investment and operational costs of photovoltaic projects, thereby enhancing the profit potential for businesses. Business management should carefully assess whether this growth is sustainable to avoid severe consequences when government subsidies are reduced or terminated. For residents, those who choose the strategy of providing locations for photovoltaic installations experience higher utility. However, with the increase in government subsidies (Fig 5(D)), residents’ utility gradually decreases. In other words, while government subsidies are on the rise, most benefits flow to businesses, and residents do not receive significant advantages. This is primarily due to changes in market supply and demand dynamics. The increase in government subsidies has not significantly increased the proportion of businesses choosing to invest in the photovoltaic industry. This is mainly because these subsidies may not be perceived as a long-term guarantee, leading businesses to maintain a cautious approach in their strategic choices. As a result, no additional businesses are entering the market, and increased market competition does not directly translate into higher rents or economic benefits for residents. It may even lead to a decline in market rents or require residents to provide more resources to maintain attractiveness. This market dynamic can decrease residents’ utility as they need to invest more resources to sustain their position in the market without necessarily receiving corresponding returns. To address this issue, the government needs to take measures to ensure that subsidies and benefits are fairly distributed among all stakeholders. This includes educating residents about the principles of the photovoltaic market and revenue generation, increasing industry transparency, and strengthening regulation to prevent fraudulent behavior by businesses. By taking these measures, the government can create an environment where all stakeholders can benefit from the growth of the photovoltaic market and contribute to a more sustainable energy future.

#### 5.3.2 The impact of government costs to solve energy consumption and pollution prevention *g*_3_

**Fig 6:**
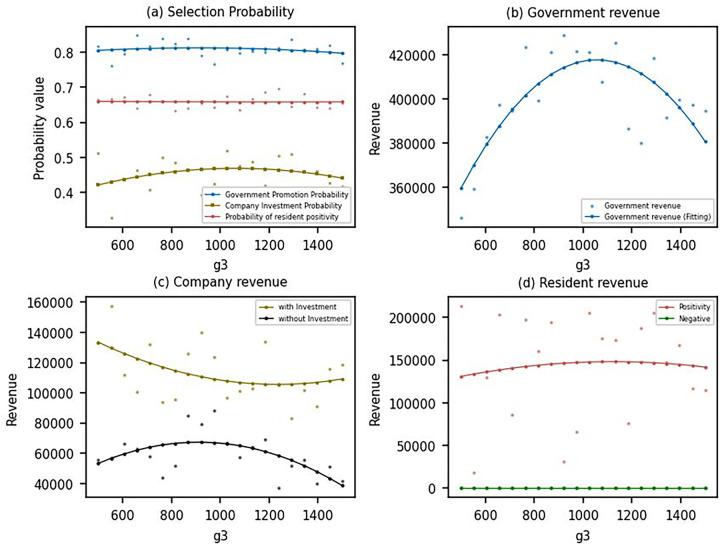
Evolutionary results under different *g*_3_.

As can be seen in Fig 6(A), the probability of the government encouraging the promotion of DPV increases as the government cost *g*_3_ to address energy consumption and pollution prevention, while there is no major change in the strategies of enterprises and residents. The government’s utility initially increases then decreases (Fig 6(B)), mainly because the government will continue to increase the promotion of DPV due to the increase in the government cost *g*_3_ to address energy consumption and pollution prevention, which makes the environmental utility offset part of the government cost *g*_3_ to address energy consumption and pollution prevention and overall makes the government’s utility increase. However, as the government cost *g*_3_ to address energy consumption and pollution prevention increases further, the increase in environmental utility is insufficient to offset the government cost *g*_3_ to address energy consumption and pollution prevention, leading to lower overall utility. As can be seen in Fig 6(C), as the government cost *g*_3_ to address energy consumption and pollution prevention increases, the returns of enterprises that adopt an investment strategy show a decreasing trend, while the returns of enterprises that adopt a non-investment strategy show an increasing trend followed by a decreasing trend. This is because if an enterprise adopts an investment strategy, it not only has to pay part of the investment but also has to bear more environmental costs because of the increase in the government cost *g*_3_ to address energy consumption and pollution prevention, which makes the enterprise’s earnings continue to decline. For an enterprise that does not invest, when the cost of solving energy consumption and pollution prevention is low, the enterprise does not have to bear too many environmental costs, so the revenue will show an increasing trend, but as this part of the cost rises, the enterprise’s revenue will continue to decline. From Fig 6(D), it can be seen that the changes in the government cost *g*_3_ to address energy consumption and pollution prevention do not significantly impact impact on the utility to residents, who continue to receive higher utility by providing sites.

#### 5.3.3 The impact of environmental protection tax *f*_3_

Environmental protection tax *f*_3_ is fine that enterprises who choose not to invest in the strategy need to pay to the government when the government is encouraging the promotion of DPV.

**Fig 7:**
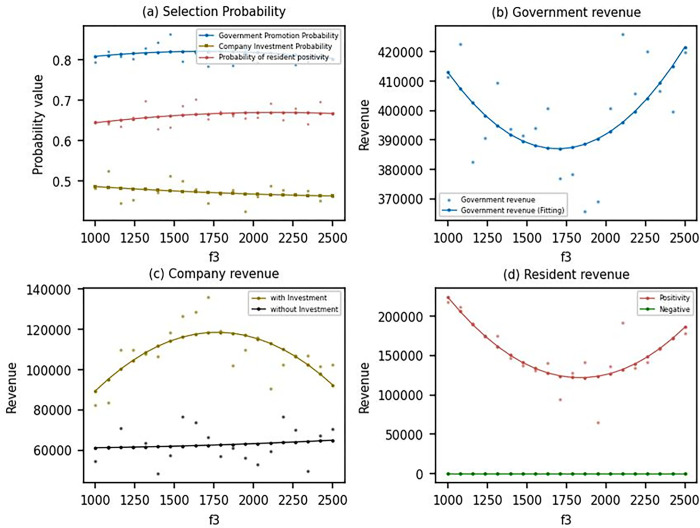
Evolutionary results under different *f*_3_.

As seen in Fig 7(A), the probability of the government encouraging promotion slightly decreases as the environmental protection tax *f*_3_ increases, while the probability of residents choosing to provide a site increases. With the increase of environmental protection tax *f*_3_, the government utility shows a decreasing and then increasing trend (Fig 7(B)). The utility of enterprises choosing the investment strategy shows an increasing and then decreasing trend, and the utility of enterprises choosing the non-investment strategy shows the opposite trend (Fig 7(C)). For residents, the utility of providing a site decreases before increasing. This trend can be mainly attributed to the fact that when the environmental protection tax *f*_3_ is low, enterprises are not incentivized to actively invest in DPV, and therefore, government utility is lower. As the environmental protection tax increases, the utility of enterprises that choose the investment strategy will continue to rise. In contrast, the utility of enterprises that choose the non-investment strategy will inevitably fall. This creates a disincentive for enterprises to opt for the non-investment strategy, promoting the adoption of DPV. As the environmental protection tax continues to increase, enterprises will increasingly take on the responsibility of promoting DPV, and the utility to residents will gradually increase. This is because the higher tax burden on enterprises that do not invest in DPV makes it more attractive for them to invest in sustainable energy sources. Consequently, the government’s promotion of DPV becomes more effective, and the increased adoption of DPV leads to higher utility for residents who provide a site. In summary, an increase in the environmental protection tax *f*_3_ encourages enterprises to invest in DPV by making the non-investment strategy less attractive. This, in turn, contributes to the promotion of DPV and increases the utility for residents who choose to provide a site for DPV projects.

#### 5.3.4 The impact of social learning effect *β*

**Fig 8:**
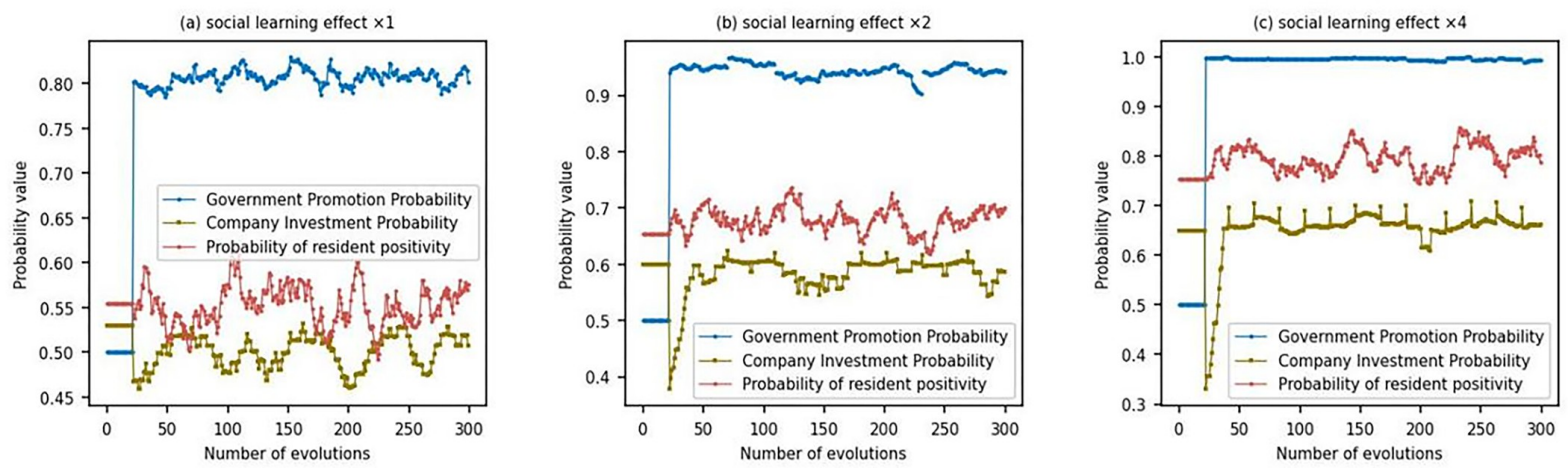
Evolutionary results under different social learning effect.

In order to investigate the impact of social learning on simulation results, we tested the results of different social learning effects. When the social learning effect is significant, each agent’s sensitivity to compensation differences increases, making it easier to learn and imitate the strategies of other agents. Through multiple simulation experiments, we have found that social learning mainly affects the strategy selection process of each subject, while its impact on returns is relatively limited. Therefore, this article focuses on showcasing the changing process of strategy choices among various subjects under the effects of 1, 2, and 4 times social learning, as follows:

As can be seen from the figure above, when the social learning effect is low (Fig 8(A)), The strategic choices of the government, enterprises, and residents undergone significant fluctuations. This indicates that in the absence of effective social learning mechanisms, there is a certain degree of blindness and uncertainty in the promotion, investment, and provision of distributed photovoltaic power generation sites by all parties. Among them, the proportion of governments choosing to encourage the promotion of distributed photovoltaic power generation has fluctuated between 0.75 and 0.85, reflecting the fluctuation of the government’s determination and intensity in promoting distributed photovoltaics. The proportion of companies choosing to invest in distributed photovoltaics has fluctuated between 0.4 and 0.55, indicating that companies have certain hesitations and doubts about investing in distributed photovoltaics. The proportion of residents providing sites fluctuates between 0.5 and 0.6, indicating that residents’ acceptance of distributed photovoltaics also fluctuates to some extent. However, with the doubling of social learning effects (Fig 8(B)), the proportion of governments choosing to encourage the promotion of distributed photovoltaic power generation increased to around 0.95, the proportion of enterprises choosing to invest in distributed photovoltaics fluctuated around 0.6, and the proportion of residents providing sites fluctuated between 0.6 and 0.7. This indicates that with the increase in social learning effects, all parties have a deeper understanding and awareness of distributed photovoltaic power generation, thus making more rational strategic choices. In addition, with the further enhancement of social learning effects (Fig 8(C)), the proportion of governments, enterprises, and residents choosing to actively support distributed photovoltaics has further increased. It can be seen that as the effect of social learning increases, the fluctuations in the strategic choices of all parties tend to stabilize, which further demonstrates that the increase in the effect of social learning not only promotes the promotion and application of distributed photovoltaic power generation but also facilitates communication and coordination among all parties to achieve resource sharing and complementary advantages.

#### 5.3.5 The impact of simultaneous changes in government subsidies and carbon taxes

**Fig 9:**
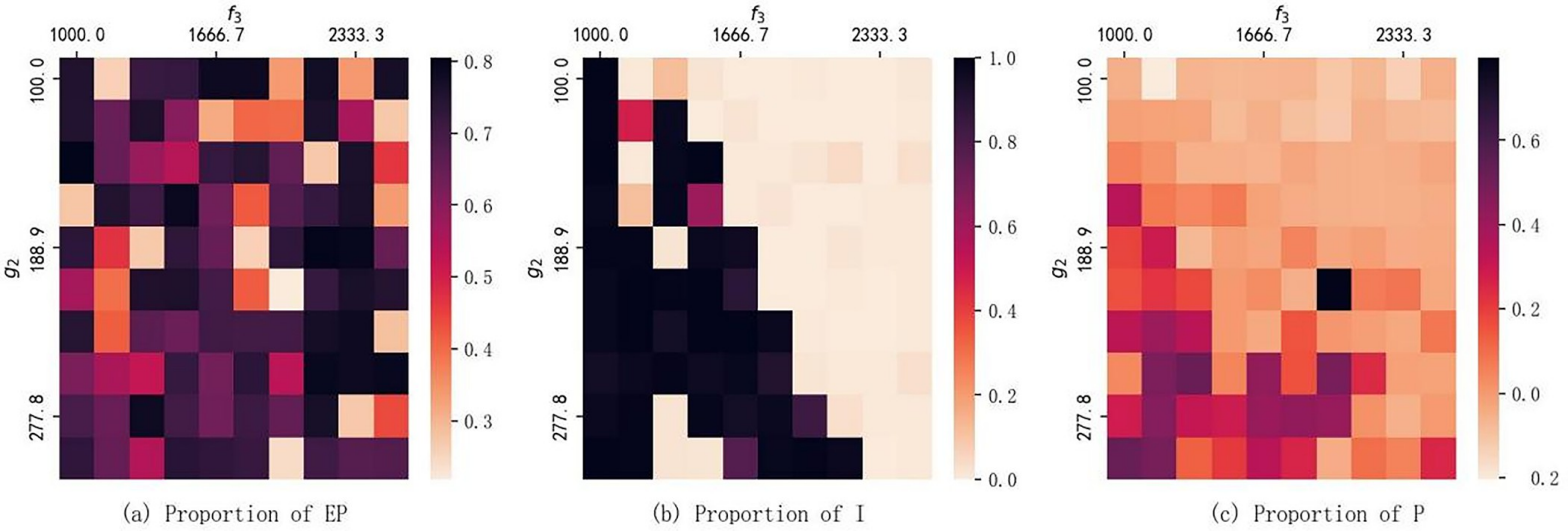
The combined impact of taxes and subsidies.

To delve into the comprehensive impact of model parameters, this paper explores the interaction between Government Subsidies and Environmental Protection Tax. Parameters such as Environmental benefits to the government and Investment costs are objectively determined by real-world data and cannot be influenced by subjective factors. Therefore, this paper focuses solely on the interplay between government-controllable parameters–subsidies and taxation. As depicted in Fig 9, the paper illustrates the percentage distribution of government strategy EP (Fig 9(A)), corporate strategy I (Fig 9(B)), and resident strategy P (Fig 9(C)) for different parameter combinations ranging from Government Subsidies 100 to 300 and Environmental Protection Tax 1000 to 2500.

The chart reveals that, for the government, subsidies and taxation have a limited impact on its decision to encourage DPV. In this decision-making process, the government needs to carefully balance the relationship between subsidies and taxation to encourage businesses and residents to invest in and provide sites for DPV. For businesses, the decision to invest in DPV is influenced by both subsidies and taxation. With an increase in subsidies, the proportion of choosing to invest in DPV gradually rises; conversely, with an increase in taxation, the proportion decreases. Clearly, when taxation reaches a certain level, the increased subsidies become insufficient to compensate for the company’s earnings, leading to a reluctance among businesses to choose DPV investments. As for residents, the proportion of choosing to provide sites gradually increases with the increase in subsidies, while it decreases with the rise in taxation. However, the changes in investment and taxation have a minor impact on residents. Residents’ sensitivity in choosing to provide sites ranges from 0.2 to 0.7, much lower than that of businesses. This provides valuable insights for the government in designing comprehensive energy policies and for businesses in formulating strategic decisions in the DPV sector. It underscores the importance of balancing subsidies and taxation in policy-making to promote sustainable energy investments.

## 6 Conclusions and implications

Driven by concerns about climate change, resource depletion, and the demand for sustainable energy, DPV systems have emerged as a promising solution. However, widespread adoption faces high upfront costs, regulatory barriers, and market uncertainties. Governments worldwide have implemented incentives to encourage DPV deployment, but their effectiveness depends on the complex interactions among stakeholders, including governments, investment enterprises, and residents. Understanding these relationships is crucial for designing effective policies that promote DPV adoption and contribute to a sustainable energy future.

This study focuses on the government, investment enterprises, and residents as the main stakeholders, considering the influence of different actors’ strategies on promoting DPV. A three-party evolutionary game model is constructed within a social network to examine these relationships. The research reveals several key findings:

(1) At the game equilibrium, both the government and residents demonstrate a proactive attitude toward promoting DPV. This indicates that their strategies align with the goals of encouraging renewable energy sources and moving towards more sustainable energy consumption patterns.

(2) Investment enterprises tend to exploit market forces and information asymmetry to secure a large portion of government subsidies. This results in an unintended consequence where increasing government subsidies do not necessarily benefit the residents. The challenge for policymakers is to design subsidy schemes that effectively target and benefit the intended recipients, ensuring that the promotion of DPV achieves its desired impact on residential stakeholders.

(3) Increasing energy consumption and pollution control costs can stimulate investment in DPV. As the financial burden of traditional energy sources and pollution management grows, companies are more likely to consider investing in sustainable energy solutions, such as DPV. This dynamic can lead to a more rapid and widespread adoption of DPV, ultimately contributing to the overall transition towards renewable energy.

(4) An increase in environmental protection taxes can, to some extent, help enterprises assume the responsibility of promoting DPV. This tax strategy can relieve the pressure on the government to promote DPV while also enhancing the benefits for residents. By imposing higher environmental taxes, the government can create a financial incentive for companies to invest in DPV projects, thereby increasing the rate of adoption and bringing greater benefits to residential stakeholders.

The above research findings also provide some management implications for policymakers:

(1) It is clear that a comprehensive approach to promoting DPV must take into account the complex interplay of strategies and interests among the government, investment enterprises, and residents. Policymakers should encourage cooperation and coordination among stakeholders to achieve common sustainable development goals.

(2) To ensure that government subsidies are effectively utilized and maximize benefits to the population, policymakers need to design more precise subsidy schemes. This requires differentiated policies for different stakeholders.

(3) Policymakers can raise the cost of traditional energy sources through measures such as environmental protection taxes, which can motivate businesses to think more about investing in sustainable energy solutions like DPV.

(4) Efforts should be made to address information asymmetry and market exploitation, ensuring that the benefits of DPV promotion are more equitably distributed among stakeholders. This could include the development of educational campaigns, public awareness initiatives, and increased transparency in the photovoltaic market.

Finally, future research could explore how the strategies and relationships among different stakeholders may evolve as the market for DPV matures. This could provide valuable insights into the long-term impacts of policy interventions and market dynamics on the promotion of DPV and the broader transition to sustainable energy systems.

## Supporting information

S1 File Data involved in this simulation can be found in the supporting information. (ZIP)
